# Integrated multi-omics and machine learning identify core mechanobiology-associated immune genes in pulmonary arterial hypertension

**DOI:** 10.3389/fimmu.2026.1867114

**Published:** 2026-10-06

**Authors:** Pei Wang, Fangchao Zhao, Ting Yang, Yadong Yuan

**Affiliations:** 1 Department of Respiratory and Critical Care Medicine, The Second Hospital of Hebei Medical University, Shijiazhuang, Hebei, China; 2 Department of Emergency, The Second Hospital of Hebei Medical University, Shijiazhuang, Hebei, China; 3 Department of Thoracic Surgery, The First Affiliated Hospital of Soochow University, Suzhou, Jiangsu, China; 4 Department of Thoracic Surgery, The Second Hospital of Hebei Medical University, Shijiazhuang, Hebei, China; 5 Department of Gastroenterology, The Fifth Clinical Medical College of Shanxi Medical University, Taiyuan, Shanxi, China

**Keywords:** diagnostic biomarker, immune-stromal interaction, machine learning, mechanobiology, pulmonary arterial hypertension

## Abstract

**Background:**

Pulmonary arterial hypertension (PAH) involves vascular remodeling and altered mechanical signaling. The immune-associated transcriptomic changes accompanying this environment remain incompletely characterized.

**Methods:**

We integrated bulk lung (GSE15197), PBMC (GSE33463), and single-cell RNA-seq (GSE169471) data using a curated set of 2, 537 mechanical-related genes. WGCNA, LASSO, random forest, and single-cell localization supported candidate prioritization. The four-gene panel was examined in a monocrotaline-induced PH rat model and PBMCs from 10 IPAH patients and 10 controls.

**Results:**

PAH transcriptomes showed enrichment of stress-fiber, actomyosin, ECM, and immune-related programs. CD83, CD86, IL6, and TLR7 were prioritized, with compartment-dependent expression patterns. Single-cell data localized CD83/CD86 to myeloid/APC compartments and IL6 to endothelial/fibroblast populations; TLR7 was sparsely detected. In MCT-treated rat lungs, CD86 and TLR7 proteins increased, whereas CD83 and IL-6 decreased. Clinical PBMCs showed corresponding expression directions, and each gene had an AUC >0.7. The exploratory four-gene disease-state score achieved an internal cross-validated AUC of 0.765.

**Conclusions:**

The four candidates provide a cellular and molecular context for investigating immune-stromal interactions in PAH, including responses to noninfectious vascular injury. The panel showed moderate internal discrimination. These findings support further clinical and functional investigation but do not establish direct mechanotransduction targets or a causal pathway linking mechanical stress to vascular inflammation.

## Introduction

Pulmonary arterial hypertension (PAH) is a progressive pulmonary disease characterized by pulmonary vasoconstriction and remodeling, affecting approximately 15 to 50 people per million worldwide (1). Its pathogenesis is primarily associated with endothelial dysfunction and abnormal proliferation of fibroblasts and smooth muscle cells (2). Vasoconstriction, vascular remodeling, pulmonary circulatory endothelial dysfunction, inflammatory responses, oxidative stress, cardiac fibrosis, pathological right ventricular remodeling, cellular hypoxia, metabolic imbalance, and thrombosis collectively contribute to the pathophysiological process of PAH (3). Although the introduction of PAH-specific treatment regimens has improved median survival in IPAH patients, long-term survival outcomes remain unsatisfactory (4). The core reasons for this lie in the complex pathogenesis of PAH, which has not yet been fully elucidated, as well as the lack of precise early-diagnostic biomarkers and effective therapeutic targets. Terminology clarification: Pulmonary hypertension (PH) refers to the broad spectrum of disorders characterized by elevated pulmonary arterial pressure, including five clinical subgroups; pulmonary arterial hypertension (PAH) specifically represents Group 1 PH driven by intrinsic pulmonary arteriopathy; idiopathic pulmonary arterial hypertension (IPAH) is a subtype of PAH without identifiable secondary etiologies.

The involvement of immune genes does not, by itself, imply an infectious cause of PAH. The clinical classification includes HIV-associated and schistosomiasis-associated PAH as well as idiopathic and other noninfectious forms (1). Innate immune responses can also be initiated by endogenous signals released during tissue injury, as illustrated by experimental evidence linking the damage-associated molecule HMGB1 and TLR4 to pulmonary vascular remodeling (5). We therefore examined immune-associated candidates in the context of vascular injury and altered mechanics, without presuming an infectious trigger.

Mechanical signaling has a profound impact on mechanical sensing, developmental processes, stem cell fate regulation, and even tumorigenesis in muscles, cells, and organs (6), playing a particularly critical role in the initiation and repair of tissue injury. Conversely, dysregulation of repair mechanisms or repeated injury may lead to pathological tissue remodeling and fibrosis (7). Due to its unique respiratory dynamics and structural characteristics, the lung microenvironment exhibits complex mechanical properties, making the lung highly susceptible to functional dysregulation (8). Studies have shown that abnormal mechanical stress is a key pathogenic factor in the onset and progression of PAH (9). Abnormal hemodynamic changes within pulmonary arterioles lead to increased mechanical stress on the vascular wall, which in turn causes significant compositional and structural changes in the extracellular matrix (ECM)—characterized by increased stiffness—ultimately reshaping the mechanical microenvironment of the cells it encloses (10). Pulmonary vascular cells can respond to ECM stiffness through YAP/TAZ-dependent signaling, linking mechanical cues to matrix remodeling (10). In this study, the mechanical-related gene (MRG) universe includes ECM components, cytoskeletal genes and mechanosensing-related signaling genes. Membership in this broad gene set does not imply that every member is a direct mechanosensor or a causal regulator of PAH. However, current PAH research largely focuses on vasoactive factors and signaling pathways, while studies on the molecular subtyping, prognostic assessment, and targeted intervention of MRGs remain scarce. The specific molecular mechanisms by which abnormal mechanical signaling regulates PAH progression have not yet been fully elucidated.

High-throughput transcriptomics and bioinformatics provide efficient tools for identifying disease-associated genes and molecular heterogeneity (11–13). Rather than applying these methods as an undifferentiated list, we organized the analysis so that each method addresses a specific question. Because mechanical forces act through a defined set of microenvironmental, cytoskeletal and mechanosensing genes, we first assembled an *a priori* mechanobiology-informed gene universe to focus the search. We then compared PAH and control transcriptomes at both bulk-tissue and single-cell resolution to establish where mechanical dysregulation occurs. To place candidate genes within coordinated expression structure rather than treating them in isolation, we applied weighted gene co-expression network analysis (WGCNA) (11). To rank candidates transparently without overstating a diagnostic model, we used machine-learning prioritization with explicit stability and interpretation steps (12). Finally, single-cell analysis (13) and signature-stratified cell-cell communication were used to resolve the cellular context of the prioritized panel. This mechanobiology-informed, hypothesis-generating strategy aims to nominate immune-associated candidates operating within the mechanically active PAH program for subsequent focused validation, rather than to deliver a finished data-driven classifier.

## Materials and methods

### Public data acquisition

Bulk lung transcriptomic data were obtained from the GSE15197 dataset (Agilent GPL6480 platform). The analytic cohort comprised 18 PAH lung samples and 13 non-diseased control lungs. Eight samples annotated as pulmonary hypertension secondary to idiopathic pulmonary fibrosis were excluded prior to differential analysis to prevent confounding from biologically distinct disease etiologies. PBMC transcriptomic data were retrieved from GSE33463, serving as an independent layer for cross-tissue directionality assessment, including 41 healthy controls and 30 idiopathic PAH (IPAH) samples. Human lung scRNA-seq data were obtained from GSE169471. Following quality-controlled preprocessing, a total of 38, 401 cells were retained for downstream analyses, comprising 25, 100 control cells and 13, 301 IPAH cells.

For microarray data, probe identifiers were mapped to official gene symbols according to the platform annotation. In instances of multiple probes mapping to the same gene, expression values were collapsed to a gene-level matrix using the maximum expression value to avoid probe-collapsing drift across analyses. A single, consistent gene-level expression matrix was used for all four-gene panel analyses to ensure reproducibility, as inconsistent probe summarization can alter CD86 and TLR7 values in this specific dataset. All subsequent differential expression, diagnostic AUC, and cross-layer analyses were therefore performed using this unified gene-level matrix.

### Mechanical-related gene universe

A curated universe of mechanical-related genes (MRGs) was assembled from three mechanobiology-oriented categories based on Gene Ontology (GO) annotations and literature review: (1) mechanical microenvironment (MM), encompassing extracellular matrix (ECM) components and remodeling enzymes; (2) cytoskeleton and force generation (CF), including actin-binding proteins and motor proteins; and (3) mechanosensing signaling (MS), covering mechanoreceptors, ion channels, and downstream signal transducers. After de-duplication, the final MRG universe comprised 2, 537 unique genes, with 1, 251 MM genes, 1, 129 CF genes, and 422 MS genes. These three categories were retained as *a priori* mechanobiology strata to ensure the mechanical signal was represented across multiple related biological dimensions rather than a single arbitrary gene list.

### Differential expression and gene set enrichment analysis

Differential expression analysis for the GSE15197 bulk dataset was performed using the `limma` R package. PAH and control samples were modeled as the primary comparison. Genes were considered differentially expressed (DEGs) if they met the study-defined thresholds (adjusted P < 0.05 and |log2 fold change| > 0.585). This yielded 4, 398 significant bulk DEGs, including 2, 571 up-regulated and 1, 827 down-regulated genes in PAH lungs.

For the GSE169471 scRNA-seq data, differential expression between IPAH and control cells was performed using the Wilcoxon rank-sum test implemented in the `Seurat` package (v4.0). Significant single-cell DEGs (adjusted P < 0.05, |log2FC| > 0.25) numbered 2, 793, with 702 up-regulated and 2, 091 down-regulated genes.

Gene set enrichment analysis (GSEA) was conducted on the ranked single-cell differential expression list using the `clusterProfiler` R package, with a specific focus on mechanical-force-related terms. The top enriched programs included stress-fiber organization, actomyosin and contractile-actin programs, ECM structural constituent activity, and basement-membrane-related processes. Enrichment plots were generated for representative mechanical programs, and intersections between DEGs and the MM, CF, and MS categories were visualized using Venn diagrams and UpSet plots.

### Identification of highly variable mechanical genes and functional network analysis

Within the overlap between DEGs and MRGs (DEG-MRG), highly variable genes (HVGs) were selected separately for the MM, CF, and MS categories using a Brennecke-type mean-variance model. For each candidate gene, mean expression, variance, and squared coefficient of variation (CV²) were calculated. A LOESS curve was fitted between log10(mean expression) and log10(CV²), and genes with positive residuals were considered highly variable relative to their mean expression level. To balance informativeness and figure clarity, the top variable genes per category were selected, resulting in 111 unique variable PAH-MRGs for downstream analyses.

Category-level biological process enrichment was performed for the selected HVGs and visualized using chord diagrams. Protein-protein interaction (PPI) networks among selected mechanical genes were retrieved from the STRING database (version 11.5) using Homo sapiens as the species and a combined confidence score cutoff of 400. The resulting network was clustered using the fast-greedy algorithm to identify interconnected mechanical-immune modules.

For pathway-level activity estimation, single-sample GSEA (ssGSEA) scores were calculated for six directional signatures (the up- and down-regulated components of MM, CF, and MS). Scores were compared between control and PAH samples using the Wilcoxon rank-sum test, and P values were adjusted using the Benjamini-Hochberg (BH) method for multiple comparisons.

### Weighted gene co-expression network analysis

Weighted gene co-expression network analysis (WGCNA) was performed on the bulk lung transcriptomic matrix to identify co-expression modules associated with PAH status and mechanical activity. The analysis utilized the top 5, 000 most variable genes to reduce noise while preserving broad co-expression structures. Sample clustering was first performed to inspect for outliers and disease-trait distribution. An optimal soft-thresholding power (β = 4) was selected based on scale-free topology fit (R^2^ > 0.85) and mean connectivity.

Modules were identified from the gene dendrogram using the dynamic tree-cut algorithm (minimum module size = 30) and summarized by module eigengenes. Module-trait correlations were calculated against PAH status and mechanical activity scores. Candidate core genes were defined by intersecting module genes with single-cell DEGs, and these core genes were subsequently subjected to GO enrichment analysis.

### Machine learning-based candidate prioritization and scoring

A transparent candidate-prioritization workflow was used to connect the mechanobiology screen to the experimentally examined four-gene panel. The funnel was: 2, 537 mechanobiology source genes (MM/CF/MS) -> 524 PAH-MRG overlap (DEGs ∩ MRGs) -> 111 variable PAH-MRG genes -> 10 machine-learning-stable computational candidates -> 4 experimentally examined genes (CD83, CD86, IL6, TLR7).

The 111 variable PAH-MRGs served as the feature pool for computational ranking using two independent algorithms: least absolute shrinkage and selection operator (LASSO) logistic regression (implemented with `glmnet`, 10-fold cross-validation) and random forest (implemented with `randomForest`, 500 trees). LASSO selection was repeated over 50 cross-validation runs to estimate selection frequency, and random forest feature importance was summarized by top-20 selection frequency. Genes consistently selected by both algorithms and showing bootstrap stability were retained as computationally stable candidates.

Given the limited sample size of the discovery cohort (31 lung samples, 18 PAH events), the event-per-variable ratio for the 111-feature screen was approximately 0.16. Therefore, the machine learning analysis was explicitly interpreted as a candidate prioritization strategy rather than a deployable diagnostic model. The final four-gene panel was not presented as the pure output of a computational classifier, but as an experimentally anchored panel contextualized by multi-layer transcriptomic evidence.

For the four-gene logistic disease-state score, leave-one-out cross-validation (LOOCV) was employed. Model discrimination was summarized by receiver operating characteristic (ROC) curve analysis, with calculation of area under the curve (AUC), and model calibration was inspected via grouped predicted-versus-observed probability plots. Exact Shapley values were computed for the four-gene logistic model to quantify each gene’s contribution to the model-predicted probability. Per-gene diagnostic performance was evaluated separately in the GSE15197 lung dataset and GSE33463 PBMC dataset. Cross-layer direction consistency was systematically assessed across bulk lung, PBMC, and single-cell data layers.

### Single-cell cell-type annotation and four-gene localization

The GSE169471 scRNA-seq object contained eight annotated lung cell populations based on canonical marker expression: myeloid/macrophage, B/plasma, T/NK, mast, endothelial, epithelial, fibroblast, and smooth muscle/pericyte cells. Cellular composition was compared between control and IPAH groups at the annotated cell-type level. Four-gene expression (CD83, CD86, IL6, TLR7) was visualized using feature plots and violin plots, and expression frequency (percentage of cells expressing the gene) and mean expression level were summarized per cell type.

The single-cell layer was specifically used to localize the four-gene signal to cellular compartments rather than to claim cell-type-specific causality. Group-level single-cell statistics were interpreted cautiously, acknowledging the limited number of IPAH donors in the single-cell dataset.

### Signature-stratified intercellular communication analysis

To contextualize the four-gene panel within the broader cellular communication network, a per-cell module score was calculated in GSE169471 based on the average expression of CD83, CD86, IL6, and TLR7. Cells were dichotomized at the median score into signature-high and signature-low groups. Intercellular communication was then inferred separately for the two groups using the `CellChat` R package (version 1.5.0) and the CellChatDB human ligand-receptor interaction database. Inferred interaction counts, interaction strengths, differential-interaction circle plots, information-flow rankings, and differential-interaction heatmaps were generated.

This communication analysis was explicitly designed as a contextual screen rather than direct functional validation. The results served to identify the cellular communication environment associated with the four-gene module, not to establish causal intercellular mechanisms.

### Establishment of monocrotaline-induced pulmonary hypertension rat model recapitulating PAH pathological features

This study followed the Guide for the Care and Use of Laboratory Animals (1996 revision, NIH Publication No. 85–23) and was approved by the Second Hospital of Hebei Medical University. Six-week-old Sprague-Dawley rats (180–220 g) were purchased from Beijing Huafukang Biotechnology Co., Ltd. Sixteen male rats were housed in an SPF environment (12-h light/dark cycle, 25 ± 2 °C, 50% ± 5% humidity) and acclimated for 2 weeks.

Monocrotaline (MCT)-induced pulmonary hypertension (PH) rat model, a classic experimental model that recapitulates vascular remodeling and right ventricular dysfunction characteristic of PAH.Rats were randomly divided into two groups (n=6 each): MCT-treated PH group (to establish PAH-like pulmonary vascular remodeling) and vehicle control group (single subcutaneous injection of 60 mg/kg MCT (monocrotaline), Sigma-Aldrich, USA) and control group (saline injection). After 3 weeks, all rats were weighed and anesthetized with 60 ml/kg chloral hydrate for subsequent assessments of PAH-like hemodynamic and pathological alterations.

### Hemodynamic measurement, tissue collection, and histopathological analysis

To evaluate right ventricular systolic pressure (RVSP) in rats, a polyethylene catheter was gently advanced into the right ventricle through the right jugular vein and coupled with a pressure transducer to record precise hemodynamic parameters. After hemodynamic detection, all rats were euthanized, and cardiac and pulmonary tissues were rapidly isolated for subsequent experimental analyses. Right ventricular hypertrophy (RVH) was evaluated by calculating the RV/(LV+S) ratio, defined as the right ventricular weight divided by the combined weight of the left ventricle and interventricular septum.

Moreover, the right lung tissues were immediately snap-frozen in liquid nitrogen and preserved for subsequent biochemical and molecular biological assays. Partial lung specimens were fixed in 4% paraformaldehyde solution, embedded in paraffin, and cut into 4-μm continuous sections. According to standard experimental procedures, tissue sections were subjected to hematoxylin and eosin (H&E) staining to observe histopathological and morphological alterations of lung tissues.

The inner and outer diameters of pulmonary arterioles were accurately measured using Image-Pro Plus software (Media Cybernetics, USA). The percentage of arterial wall thickness was calculated via the formula: [(external diameter − internal diameter)/external diameter] × 100. This quantitative index was applied to reflect the degree of pulmonary vascular remodeling, which serves as a core pathological hallmark of PAH.

### Western blot analysis

Cells and tissue samples were lysed with RIPA lysis buffer containing protease and phosphatase inhibitor cocktails. Total protein concentration was quantified via a BCA protein assay kit. Equivalent protein samples (20–30 μg) were resolved by SDS-PAGE and electrotransferred onto PVDF membranes. Membranes were blocked with 5% non-fat dry milk diluted in TBST for 1 hour at room temperature, followed by overnight incubation with primary antibodies at 4 °C. The primary antibodies utilized in this study included: TLR7 (17232-1-AP), CD86 (26903-1-AP), CD83 (27873-1-AP), IL-6 (21865-1-AP), and α-Tubulin (80762-1-RR), all purchased from Proteintech, Rosemont, USA. After thorough washing with TBST, the membranes were incubated with HRP-linked secondary antibodies for 1 h at room temperature. Target protein bands were detected using an enhanced chemiluminescence (ECL) imaging system, and band gray values were analyzed with ImageJ software for quantitative comparison. α-Tubulin was applied as the internal loading control.

### Quantitative PCR analysis

Total RNA was extracted from PBMCs using TRIzol reagent (Sigma-Aldrich, T9424, USA), strictly adhering to the manufacturer’s guidelines. This RNA was then reverse-transcribed into first-strand complementary DNA (cDNA) employing the HiFi II 1st Strand cDNA Synthesis Kit (Yeasen, 11119ES60, China). The resulting cDNA was subjected to quantitative real-time PCR (qPCR) on an Applied Biosystems real-time PCR system, utilizing SYBR Green Master Mix (Yeasen, 11184ES03, China). Relative mRNA expression levels were calculated using the 2^-ΔΔCt^ method, with GAPDH as the internal control for normalization. All samples were run in triplicate. The primer sequences used in RT-PCR are shown in **Table 1**.

**Table 1:** Primer sequences.

Primer name	Primer sequences
CD86 Forward	TCAATGGGACTGCATATCTGCC
CD86 Reverse	GCCAAAATACTACCAGCTCACT
CD83 Forward	AGCTGCCCAAGATCAACATCC
CD83 Reverse	GGTGAAGTTGATGGTGGTGAG
TLR7 Forward	GCTGGATGGTGCTGTGTATG
TLR7 Reverse	CCAGGGTTGATGTTGTTGAGG
IL-6 Forward	GGCACTGGCAGAAAACAACC
IL-6 Reverse	GCAAGTCTCCTCATTGAATCC
GAPDH Forward	ACCACAGTCCATGCCATCAC
GAPDH Reverse	GCTTCACCACCTTCTTGATG

### Clinical sample collection and diagnostic criteria

Peripheral blood mononuclear cell (PBMC) samples were obtained from 10 patients with idiopathic pulmonary arterial hypertension (IPAH) and 10 age- and sex-matched healthy controls at the Second Hospital of Hebei Medical University between March 2025 and March 2026. The study was approved by the institutional ethics committee (approval No. 2025-Y73), and written informed consent was obtained from all participants.

All IPAH patients underwent comprehensive diagnostic evaluation according to the 2022 ESC/ERS guidelines for the diagnosis and treatment of pulmonary hypertension.

Inclusion criteria for IPAH patients were: (1) age 18–75 years; (2) confirmed diagnosis of IPAH as defined above; (3) World Health Organization functional class (WHO-FC) II–III; and (4) no change in PAH-targeted therapy within 3 months prior to enrollment. Exclusion criteria were: (1) pulmonary hypertension due to other etiologies (group 2–5); (2) active infection, autoimmune disease, or malignancy; (3) severe hepatic or renal dysfunction; and (4) pregnancy or lactation.

For healthy controls, inclusion criteria were: (1) no history of cardiovascular, pulmonary, or systemic diseases; (2) normal physical examination and routine blood tests; and (3) no long-term medication use. Exclusion criteria were the same as for IPAH patients with respect to infection, autoimmune disease, and organ dysfunction.

Demographic and clinical data, including age, sex, body mass index (BMI), WHO functional class, 6-minute walk distance (6MWD), NT-proBNP levels, and hemodynamic parameters (mPAP, PAWP, PVR, cardiac output), were collected from medical records. These data are summarized in **Table 2**.

**Table 2:** Demographic and clinical characteristics of IPAH patients and healthy controls.

Characteristic	IPAH patients (n=10)	Healthy controls (n=10)	P value
Age (years), mean ± SD	48.5 ± 12.3	47.2 ± 11.8	0.812
Sex (male/female)	3/7	4/6	0.639
BMI (kg/m²), mean ± SD	23.6 ± 2.8	24.1 ± 2.5	0.674
WHO functional class (II/III)	6/4	–	–
6MWD (m), mean ± SD	412 ± 58	–	–
NT-proBNP (pg/mL), median (IQR)	1250 (620–2480)	–	–
mPAP (mmHg), mean ± SD	48.5 ± 9.2	–	–
PAWP (mmHg), mean ± SD	9.2 ± 2.5	–	–
PVR (Wood units), mean ± SD	8.4 ± 2.6	–	–
Cardiac output (L/min), mean ± SD	4.1 ± 0.8	–	–

SD, standard deviation; IQR, interquartile range; BMI, body mass index; WHO, World Health Organization; 6MWD, 6-minute walk distance; NT-proBNP, N-terminal pro-brain natriuretic peptide; mPAP, mean pulmonary arterial pressure; PAWP, pulmonary arterial wedge pressure; PVR, pulmonary vascular resistance. P values were calculated using Student’s t-test (continuous) or chi-square test (categorical).

### Statistical analysis

All statistical analyses and data visualizations were performed using R (version 4.3.0) and Python (version 3.10.11). For continuous variables, Pearson’s correlation coefficient was used to analyze correlations between variables if the data followed a normal distribution; otherwise, Spearman’s correlation coefficient was used. Differences between groups were assessed using the Wilcoxon signed-rank test. Statistical significance was set at p < 0.05 (*p < 0.05, **p < 0.01, ***p < 0.001).

## Results

### PAH exhibits broad mechanical transcriptomic remodeling

To establish the global transcriptomic landscape, we first analyzed bulk lung data from GSE15197, identifying 4, 398 differentially expressed genes between PAH and control lungs, including 2, 571 up-regulated and 1, 827 down-regulated genes (**Figure 1A**). Parallel analysis of single-cell data from GSE169471 revealed 2, 793 DEGs, comprising 702 up-regulated and 2, 091 down-regulated genes (**Figure 1A**). These volcano plots demonstrate that PAH is accompanied by extensive transcriptional remodeling at both tissue and single-cell resolution.

**Figure 1:**
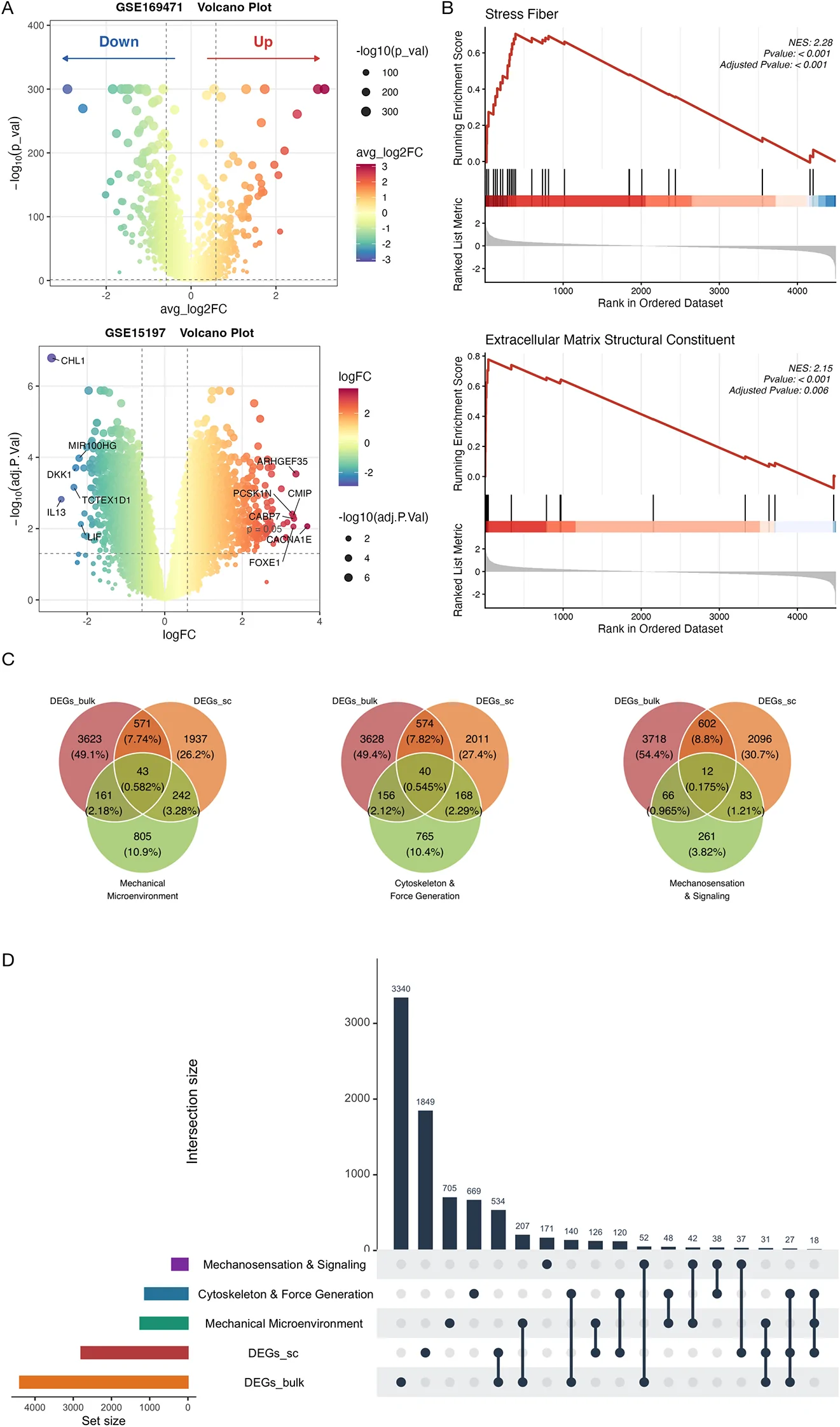
Transcriptomic landscape of mechanobiology-associated dysregulation in PAH. **(A)** Volcano plots showing differential expression in GSE169471 single-cell lung data and GSE15197 bulk lung data. **(B)** Representative GSEA plots for mechanical-force programs enriched in the single-cell ranked list. **(C)** Venn diagrams showing overlap between DEGs and the three mechanical-related gene categories: mechanical microenvironment (MM), cytoskeleton and force generation (CF), and mechanosensing signaling (MS). **(D)** UpSet plot summarizing intersections among bulk DEGs, single-cell DEGs and mechanical categories. The image establishes the mechanically dysregulated gene pool used for downstream analyses.

Gene set enrichment analysis (GSEA) of the single-cell ranking highlighted a strong enrichment of mechanical-force-related programs. Representative enriched pathways included stress-fiber organization, actomyosin structure formation, and extracellular matrix structural constituent activity (**Figure 1B**), consistent with a mechanical remodeling phenotype. Intersection analysis further demonstrated that both bulk and single-cell DEGs substantially overlapped with the MM, CF, and MS gene sets. Venn and UpSet plots summarized these intersections, indicating that PAH dysregulation spans extracellular microenvironment, cytoskeletal-force, and mechanosensing components rather than being confined to a single mechanical category (**Figures 1C, D**).

### Mechanical candidate genes show coordinated functional and network architecture

After intersecting DEGs with the mechanical-related gene universe, highly variable genes were first identified within each mechanical category. Mean-variance correction initially retained 75 MS, 208 MM, and 167 CF category-specific HVG entries. To obtain a focused candidate set for downstream visualization and prioritization, the top residual-ranked genes were then retained within each category, yielding 111 unique variable PAH-MRGs (**Figure 2A**). This step reduced the large DEG-MRG overlap to a more focused set of variable mechanical candidates.

**Figure 2:**
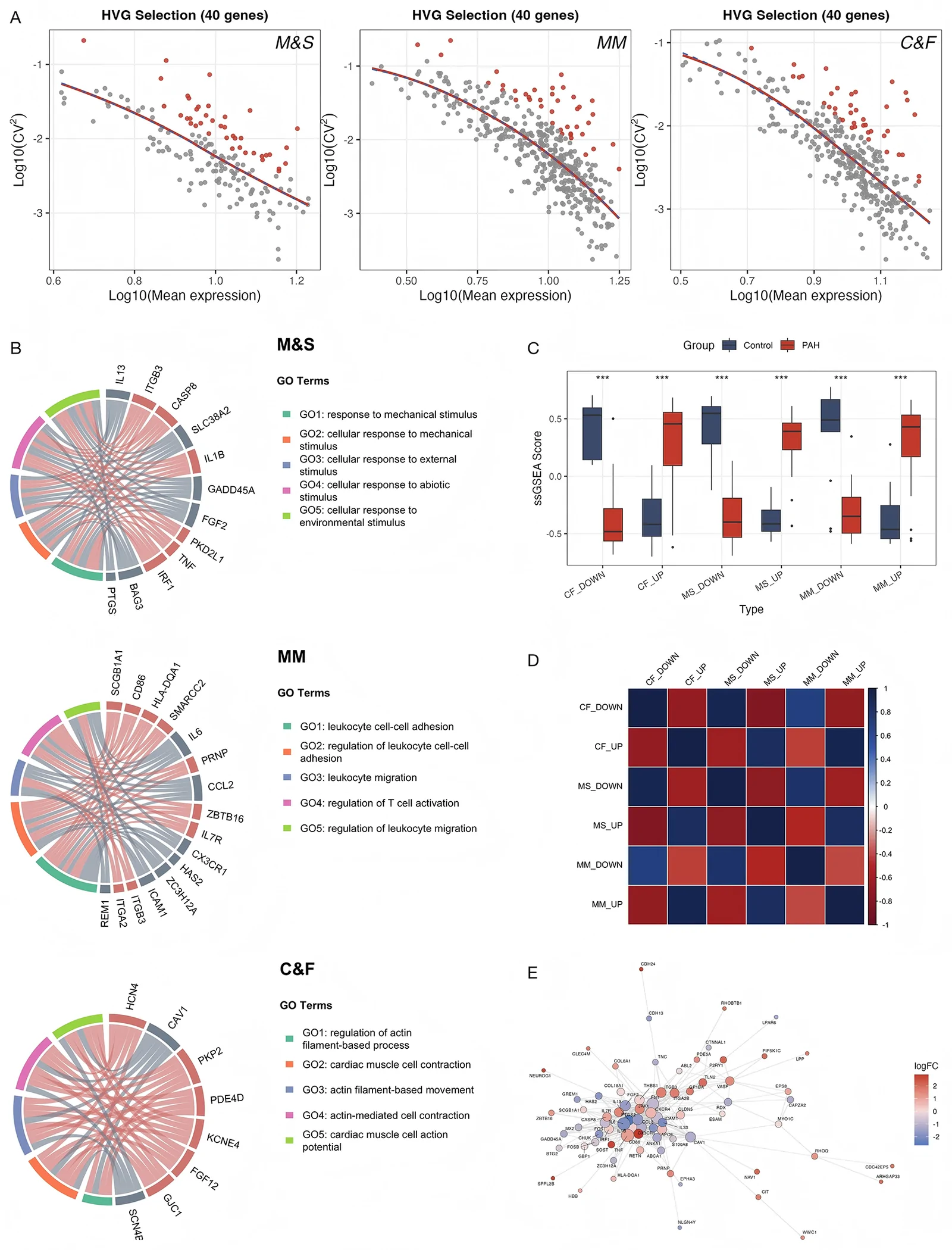
Functional landscape of variable mechanical candidate genes. **(A)** Highly variable gene selection within MM, CF and MS categories using a Brennecke-type mean-variance model. **(B)** GO chord diagrams showing biological-process enrichment for selected category genes. **(C)** ssGSEA comparison of up- and down-regulated mechanical-category signatures between control and PAH lung samples. **(D)** Correlation heatmap of mechanical-category scores. **(E)** STRING protein-protein interaction network of selected mechanical candidates, clustered by network community.

Gene Ontology chord diagrams demonstrated that the selected genes were enriched for biologically relevant mechanical and immune functions. The MS category was prominently connected to terms such as “response to mechanical stimulus, “ the CF category to “contractile actin filament bundle assembly” and “actin-mediated cell contraction, “ and the MM category to “leukocyte adhesion” and “extracellular matrix organization” (**Figure 2B**). Thus, the candidate universe retained functional coherence after stringent filtering.

Single-sample GSEA provided sample-level evidence that mechanical category activity significantly differed between PAH and control lungs. All six directional signatures (MM up/down, CF up/down, MS up/down) were significantly different after FDR adjustment, with adjusted P values ranging from 10–^4^ to 10^-6^ (**Figure 2C**). The category-correlation heatmap further revealed that up-regulated mechanical signatures were positively correlated with each other but negatively correlated with down-regulated signatures (**Figure 2D**). STRING protein-protein interaction network analysis produced a highly interconnected mechanical-candidate network rather than a sparse set of unrelated genes. Fast-greedy clustering separated the network into distinct communities containing ECM, cytoskeletal, immune-adhesion, and mechanosensing components (**Figure 2E**), supporting the interpretation that the selected variable genes represent a coherent mechanobiology-linked program.

### WGCNA recovers PAH-associated co-expression context

To determine whether the mechanical candidates are embedded within broader lung co-expression modules, we performed weighted gene co-expression network analysis. Sample clustering showed clear group structure without requiring outlier exclusion (**Figure 3A**). Soft-thresholding analysis selected a power of β = 4 (scale-free R^2^ > 0.85, mean connectivity ≈ 20), which successfully established a scale-free network topology (**Figure 3B**). Gene dendrogram cutting produced 14 distinct co-expression modules (**Figure 3C**).

**Figure 3:**
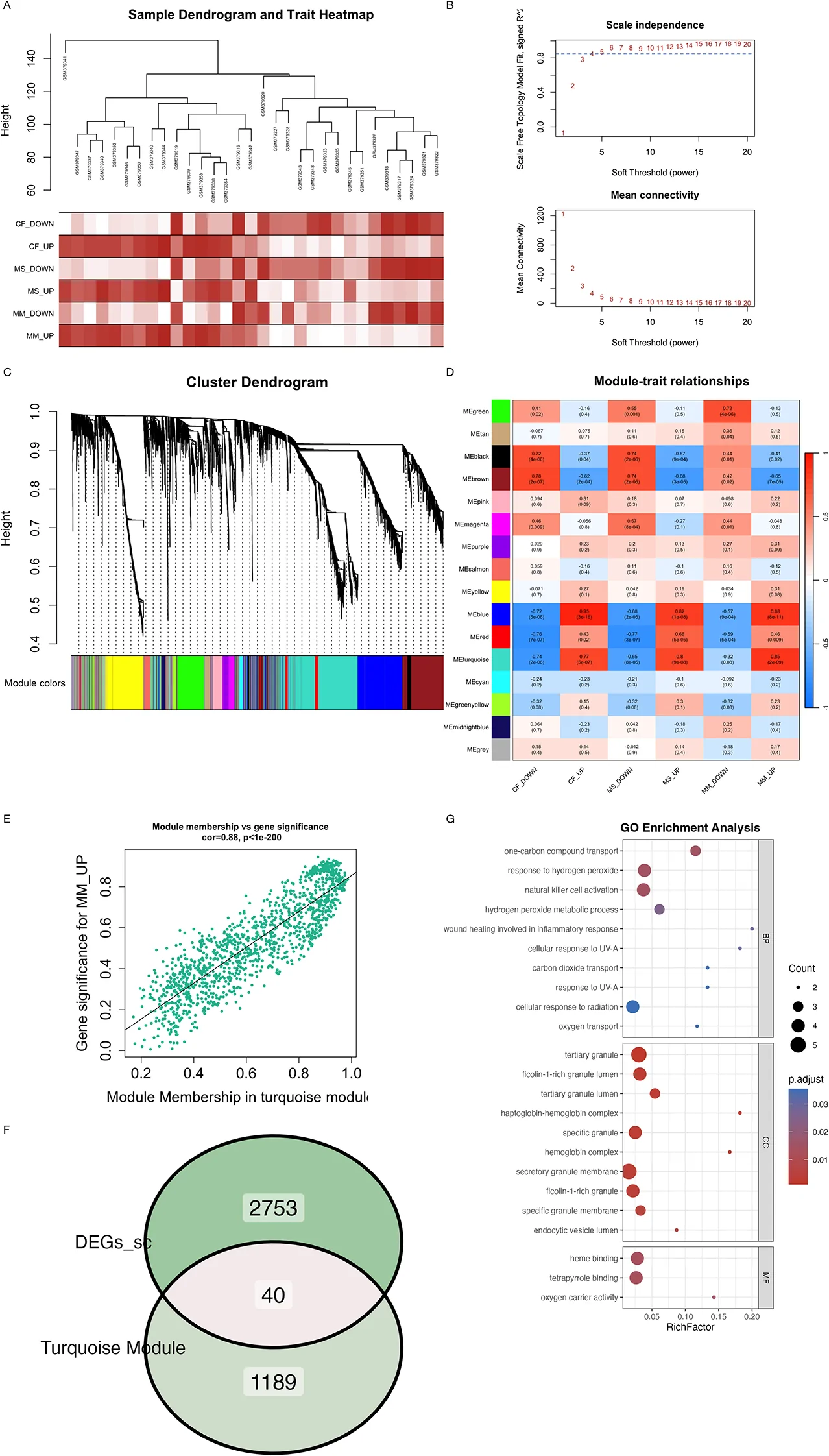
Weighted gene co-expression network analysis of PAH lung transcriptomes. **(A)** Sample clustering dendrogram with disease-trait annotation. **(B)** Soft-thresholding analysis showing scale-free topology fit and mean connectivity. **(C)** Gene dendrogram and module assignment. **(D)** Module-trait correlation heatmap linking module eigengenes to PAH status and mechanical activity. **(E)** Gene significance versus module membership for the leading module. **(F)** Venn diagram identifying 40 core genes shared between WGCNA module genes and single-cell DEGs. **(G)** GO enrichment of core module genes.

Module-trait correlation analysis linked multiple module eigengenes to PAH status and mechanical activity scores. Notably, the turquoise module showed the strongest positive correlation with the CF-related mechanical score (correlation = 0.68, P = 2e-04), while the green module was significantly correlated with MM and MS scores (**Figure 3D**). Gene significance versus module membership analysis demonstrated that genes within the turquoise module displayed coordinated module membership and disease/mechanical association (correlation = 0.877, P < 1e-200, **Figure 3E**).

Core module genes, defined by the intersection of WGCNA module genes and single-cell DEGs, comprised 40 genes (**Figure 3F**). GO enrichment analysis of these core genes (**Figure 3G**) was dominated by heme, oxygen-carrier and gas-transport terms (heme binding, tetrapyrrole binding, oxygen-carrier activity, hemoglobin and haptoglobin-hemoglobin complexes, one-carbon compound transport and carbon-dioxide transport), oxidative-stress responses (response to hydrogen peroxide, hydrogen-peroxide metabolic process and cellular response to UV-A) and innate-immune and granulocyte programs (natural killer cell activation, wound healing involved in inflammatory response, and tertiary, specific and ficolin-1-rich granule compartments). This oxygen-handling, oxidative-stress and innate-inflammatory profile is biologically coherent for PAH, consistent with the central roles of hypoxia and oxidative stress in pulmonary vascular disease. WGCNA was therefore used as contextual support for the mechanical-immune background of the study, not as the independent method that selected the final four-gene panel.

### Candidate gene prioritization

The candidate funnel reduced the analysis from the full mechanical source universe to an experimentally examined four-gene panel. Specifically, the workflow moved from 2, 537 source genes to 524 PAH-MRG overlap genes, 111 variable PAH-MRG genes, 10 machine-learning-stable computational candidates, and finally four genes with experimental expression-validation support (CD83, CD86, IL6, and TLR7) (**Figure 4A**). LASSO coefficient paths demonstrated that the regularization process progressively eliminated less informative genes, while random forest analysis identified a stable subset of high-importance features (**Figure 4B**). Among the machine-learning-stable candidates, CD83, CD86, IL6 and TLR7 were retained because they simultaneously satisfied three criteria: differential expression in PAH lung, recovery by both LASSO and random-forest stability selection, and availability of orthogonal experimental expression validation in the MCT model and patient PBMCs; the panel is thus an experimentally anchored subset of the stable computational candidates rather than the raw output of a purely computational classifier.

**Figure 4:**
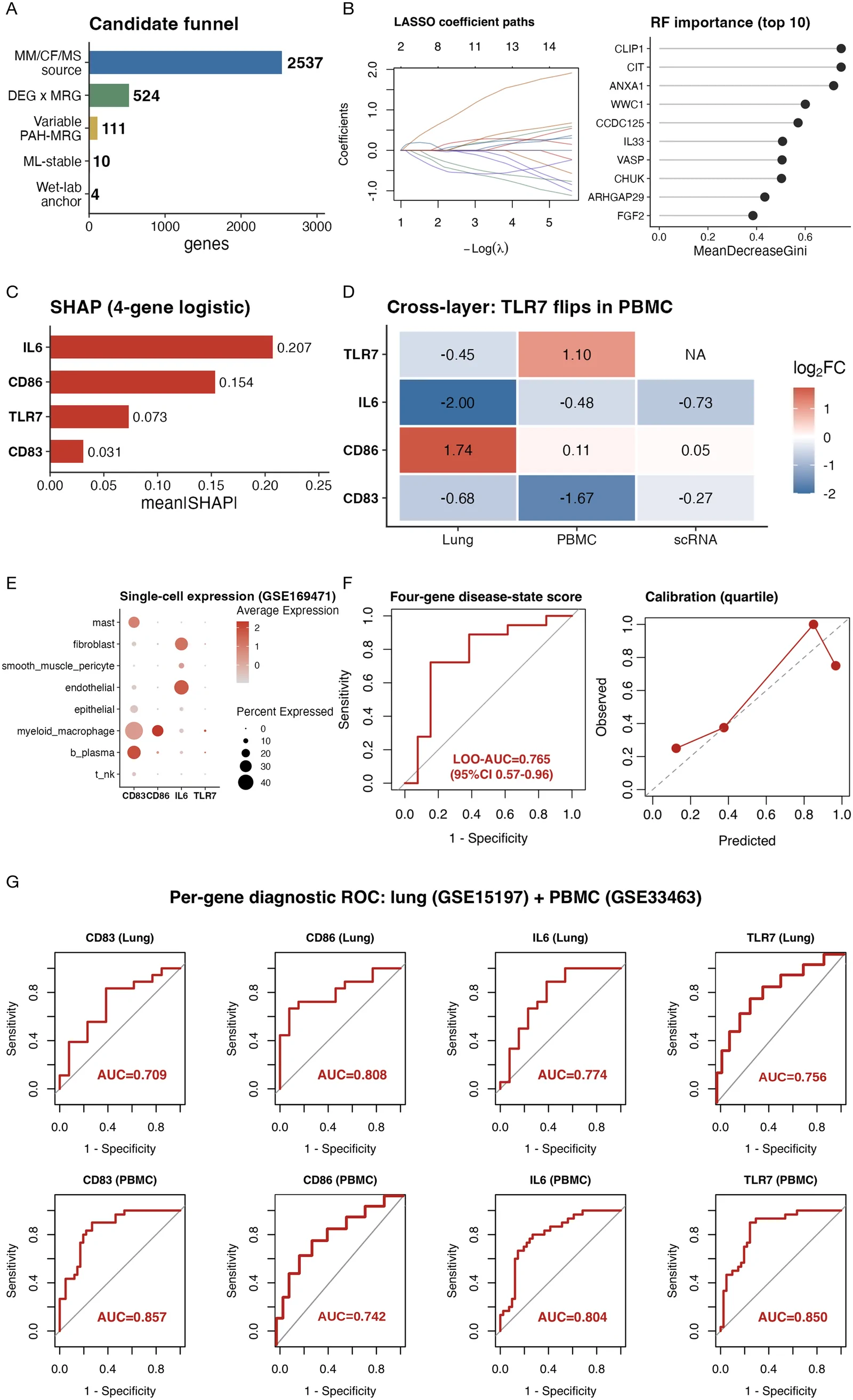
Transparent candidate prioritization and diagnostic value of the experimentally examined four-gene panel. **(A)** Candidate funnel from mechanical source genes to PAH-MRG overlap, variable PAH-MRG genes, machine-learning-stable candidates and the four-gene panel. **(B)** LASSO coefficient paths and random-forest importance. **(C)** Shapley contribution of CD83, CD86, IL6 and TLR7 in the four-gene logistic model. **(D)** Cross-layer direction of the four genes in lung bulk, PBMC and single-cell data. **(E)** Single-cell expression across cell types. **(F)** Four-gene disease-state score ROC and calibration. **(G)** Per-gene ROC curves in lung and PBMC. The ML component is presented as candidate prioritization rather than a deployable diagnostic model.

Shapley value decomposition of the four-gene logistic model revealed that IL6 and CD86 contributed the largest mean absolute Shapley values (0.207 and 0.154, respectively), whereas TLR7 and CD83 contributed less (0.073 and 0.031, respectively) (**Figure 4C**). The four-gene disease-state score showed a leave-one-out AUC of 0.765 (95% CI: 0.621–0.909), with a support vector machine sensitivity analysis yielding a concordant AUC of 0.722.

At the single-gene level, diagnostic performance varied across tissue compartments (**Figure 4D**): CD83 (lung AUC = 0.709, PBMC AUC = 0.857); CD86 (lung AUC = 0.808, PBMC AUC = 0.440); IL6 (lung AUC = 0.774, PBMC AUC = 0.804); TLR7 (lung AUC = 0.624, PBMC AUC = 0.850). Cross-layer direction analysis revealed that CD83, CD86, and IL6 showed broadly interpretable directional patterns across lung bulk, PBMC, and single-cell layers, whereas TLR7 showed context-dependent behavior. Specifically, CD83 was down-regulated in lung (logFC = -0.681, P = 0.027) and PBMC (logFC = -1.673, P = 8.48e-09); CD86 was up-regulated in lung (logFC = 1.737, P = 0.002) but showed weak PBMC diagnostic separation in the public PBMC dataset; IL6 was down-regulated in both lung (logFC = -2.001, P = 0.003) and PBMC (logFC = -0.482, P = 8.69e-06); TLR7 was down-regulated in lung (logFC = -0.448, P = 0.18) but up-regulated in PBMC (logFC = 1.099, P = 8.13e-08). The independent PBMC transfer analysis further supported this: dropping TLR7 increased the independently standardized PBMC transfer AUC from 0.590 to 0.780, confirming that TLR7 should be interpreted as a context-dependent peripheral immune signal rather than a uniform lung-bulk marker.

Single-cell expression across cell types further placed CD83 and CD86 in immune/APC-related compartments and IL6 in structural-cell compartments (**Figure 4E**). The four-gene disease-state score provided moderate discrimination and calibration in the lung discovery cohort (**Figure 4F**).

Per-gene ROC curves across lung and PBMC datasets are presented in **Figure 4G**. In the public lung and PBMC datasets, CD83, CD86, IL6, and TLR7 showed heterogeneous single-gene diagnostic performance rather than uniformly exceeding AUC 0.7; therefore, these ROC analyses were used as model-transparency and context checks, not as independent validation of deployable diagnostic biomarkers. Additional candidate-prioritization diagnostics, including univariate diagnostic performance, LASSO cross-validation behavior, and overlap between LASSO- and random-forest-selected stable genes, are provided in **Supplementary Figure S1**.

### Single-cell analysis localizes the panel to myeloid/APC and structural compartments

Single-cell annotation resolved eight major lung cell populations (**Figure 5A**). The largest compartment was myeloid/macrophage, accounting for 57.6% of control cells and 45.4% of IPAH cells. Endothelial cells increased from 7.4% in controls to 15.1% in IPAH, while mast cells increased from 1.2% to 3.9% (**Figure 5B**). Canonical marker expression (e.g., CD68 for myeloid, CD79A for B cells, CD3D for T cells, VWF for endothelial, EPCAM for epithelial, COL1A1 for fibroblasts) supported the cell-type annotation (**Figure 5C**).

**Figure 5:**
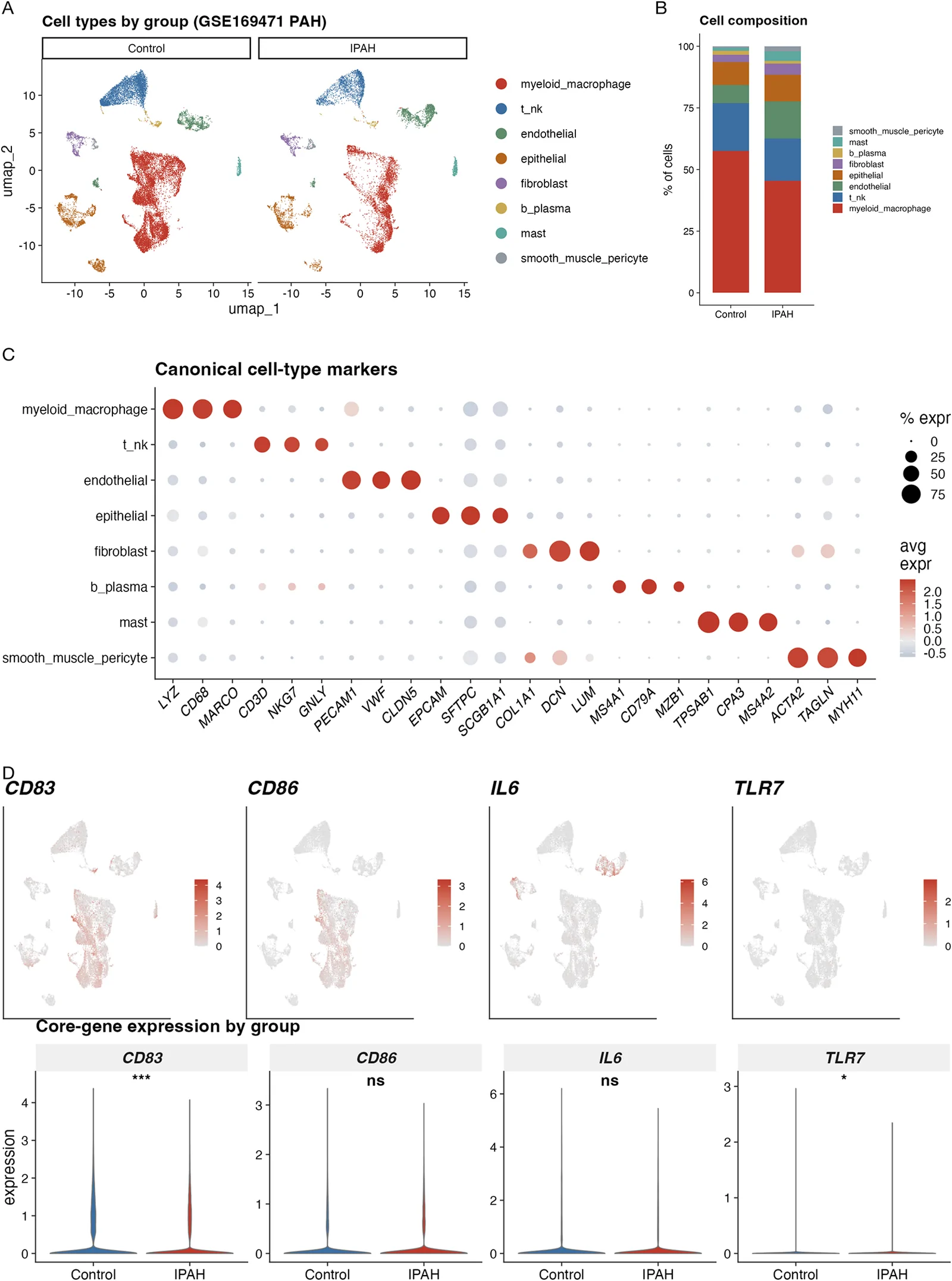
Single-cell localization of the four-gene panel. **(A)** UMAP visualization of annotated lung cell types split by control and IPAH status. **(B)** Cellular composition comparison between control and IPAH. **(C)** Canonical marker dot plot supporting cell-type annotation. **(D)** Feature plots and violin plots showing CD83, CD86, IL6 and TLR7 expression across disease groups. CD83 and CD86 localize mainly to myeloid/macrophage and antigen-presenting compartments, whereas IL6 shows structural-cell enrichment and TLR7 is sparsely detected.

The four-gene panel showed cell-context localization rather than uniform expression across all lung cell types. CD83 was detected in 47.3% of myeloid/macrophage cells and 34.4% of B/plasma cells, consistent with antigen-presenting and immune-activation contexts. CD86 was detected in 28.6% of myeloid/macrophage cells, supporting a myeloid/APC interpretation. IL6 was more evident in structural compartments, including endothelial and fibroblast populations, whereas TLR7 remained sparsely detected across the single-cell object (**Figure 5D**). When expression was stratified by disease group, CD83 showed the clearest single-cell group difference, while CD86, IL6, and TLR7 showed weaker or non-significant differences in this dataset. This compartment-specific pattern provides a biological explanation for the mixed bulk and PBMC behavior observed in **Figure 4**, especially for IL6 and TLR7. Exploratory myeloid/APC subclustering and cell-state analyses are presented in **Supplementary Figure S2**.

### Four-gene signature status defines distinct intercellular communication contexts

The four-gene module score divided cells into signature-high and signature-low groups for cell-communication analysis (**Figure 6A**). Cellular composition differed between score-defined groups: endothelial cells represented 13.0% of signature-high cells and 17.2% of signature-low cells, while myeloid/macrophage representation remained a major component in both strata (**Figures 6B, C**). This indicates that the four-gene score captures a cellular-state context rather than a single isolated cell type.

**Figure 6:**
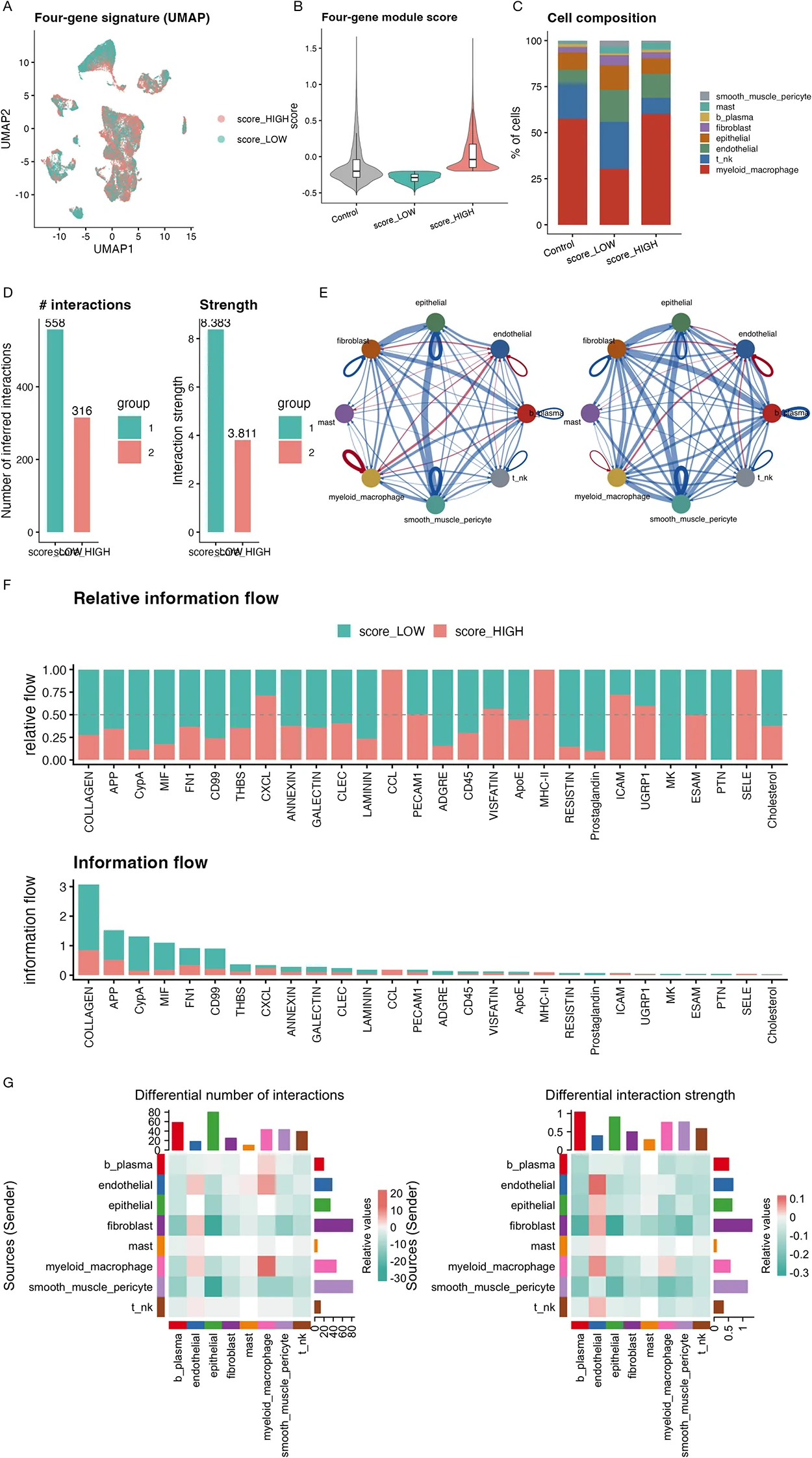
Signature-stratified cell-cell communication context. **(A)** Four-gene module-score grouping on the single-cell UMAP. **(B)** Module-score distribution across control, signature-low and signature-high cells. **(C)** Cell-type composition of the score-defined groups. **(D)** Numbers and strengths of inferred CellChat interactions. **(E)** Differential-interaction circle plots. **(F)** Relative and absolute information-flow ranking of signaling pathways. **(G)** Differential-interaction heatmaps. These data describe expression-derived communication context and are not interpreted as validated functional signaling.

CellChat analysis showed differences in inferred interaction number and strength between signature strata (**Figure 6D**). Differential-interaction circle plots summarized the direction of altered communication between major cell compartments (**Figure 6E**). Relative and absolute information-flow ranking highlighted pathways including COLLAGEN, APP, MIF, FN1, CXCL, and CD99 among the communication programs observed in the lung cell network (**Figure 6F**). The strongest communication differences involved myeloid and stromal compartments, placing the four-gene panel within a broader myeloid-structural communication background. Differential interaction heatmaps further detailed the altered ligand-receptor pairing between signature-high and signature-low cells (**Figure 6G**). Because CellChat infers putative ligand-receptor interactions from expression data, these results are interpreted as communication-context evidence rather than validated signaling function.

### Establishment of an MCT-induced PH rat model

We successfully established a rodent pulmonary hypertension (PH) model that recapitulates typical PAH pathological lesions. Compared with the control group, the right ventricular systolic pressure (RVSP) in the PAH model group was significantly increased (**Figure 7A**). In addition, the degree of right ventricular hypertrophy (RVH) was also significantly increased, which was specifically reflected by the elevation of the right ventricle/(left ventricle + septum) (RV/LV+S) ratio, and the ratio in rats of the MCT-treated group was significantly higher than that in the control group (**Figure 7B**). Pulmonary arterial medial thickness was greater in MCT-induced PH rats than in control rats (**Figure 7C**). Subsequently, we detected the protein expression levels of target genes in lung tissues of PH rats with PAH phenotypes. The results showed that CD86 and TLR7 genes were significantly upregulated in the lung tissues of PH model rats, while CD83 and IL-6 genes were significantly downregulated in the lung tissues of PH model rats (**Figure 7D**).

**Figure 7:**
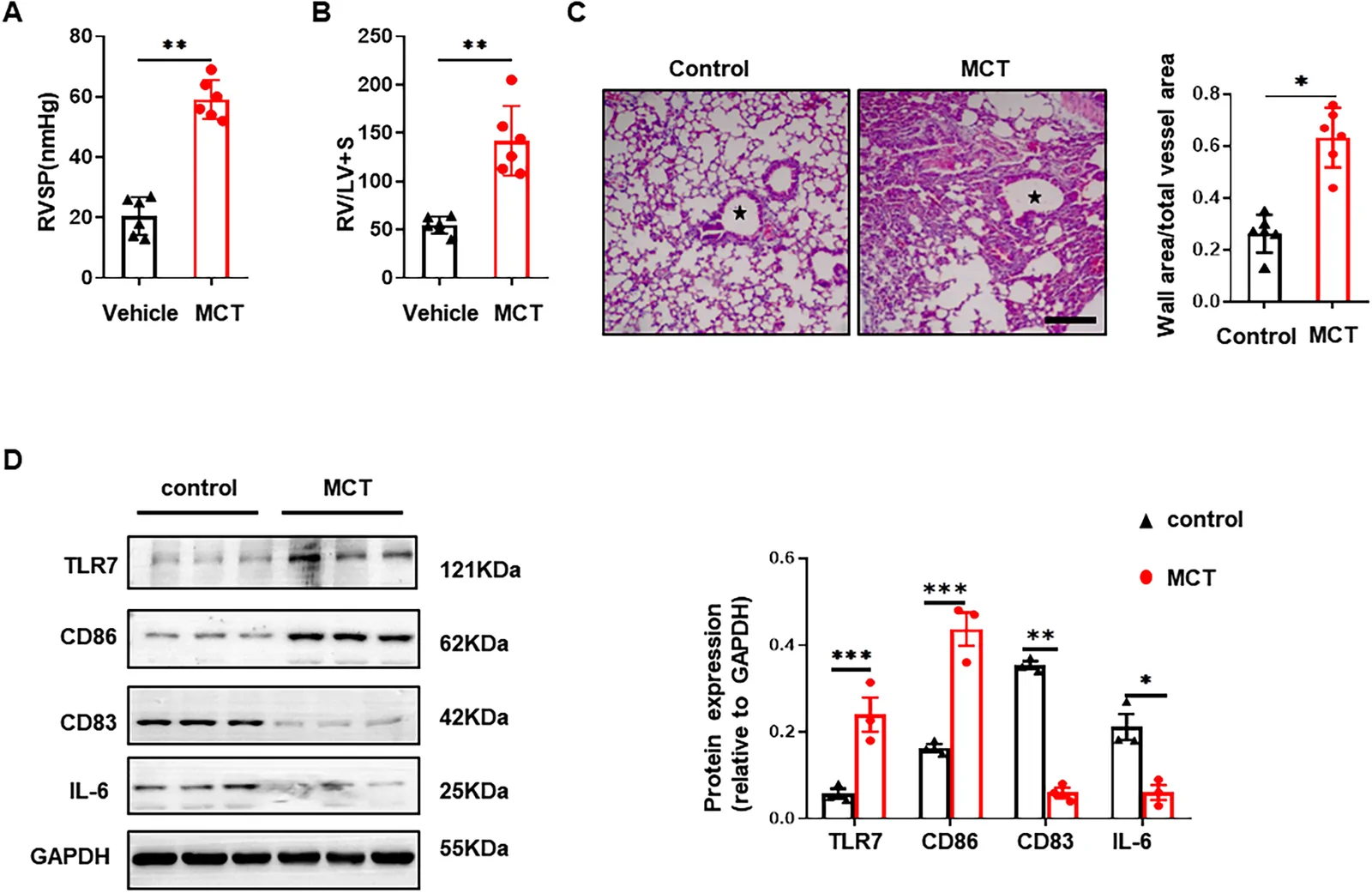
Establishment of MCT-induced pulmonary hypertension (PH) rat model recapitulating PAH pathological features. **(A)** RVSP and **(B)** RV/(LV+S) were detected in MCT-induced pulmonary hypertension (PH) rats and control rats (n=6/group). **(C)** Representative H&E staining images of lung tissues from the two groups. Black asterisks mark the pulmonary artery lumen. Scale bar = 100 μm (n=6/group). **(D)** Representative Western blot bands and quantitative analysis of CD86, TLR7, CD83, IL-6, and GAPDH in lung tissues of MCT-induced PH rats and control rats. Data represent mean ± SEM. *P < 0.05; **P < 0.01; ***P < 0.001 compared to control, as analyzed by unpaired t test or Mann-Whitney test as appropriate.

### Validation of the four immune biomarkers in PH rats and IPAH patient PBMCs

Based on the above bioinformatics analysis and experimental verification results, we confirmed that CD86, TLR7, CD83, and IL-6 can serve as marker genes reflecting PAH-related pathological changes in this PH rat model, so they were selected for subsequent experimental verification. First, we collected peripheral blood mononuclear cells (PBMCs) from 10 patients with idiopathic pulmonary arterial hypertension (IPAH) and 10 healthy controls. The detection results showed that the expression concentrations of CD83 and IL-6 in IPAH patients were significantly lower than those in healthy controls, while the expression concentrations of CD86 and TLR7 were significantly higher than those in healthy controls (**Figure 8A**). The diagnostic efficacy of CD86, TLR7, CD83, and IL-6 for PAH was further evaluated by ROC curve analysis. The results showed that the AUC values of the above four genes were all greater than 0.7, indicating that CD86, TLR7, CD83, and IL-6 show candidate diagnostic potential for IPAH in this small validation cohort (**Figure 8B**). Collectively, these findings demonstrate that CD86, TLR7, CD83, and IL-6 are associated with IPAH disease status in this small cross-sectional cohort and show candidate diagnostic potential, supporting further validation of their diagnostic relevance while any therapeutic interpretation remains hypothesis-generating pending functional studies.

**Figure 8:**
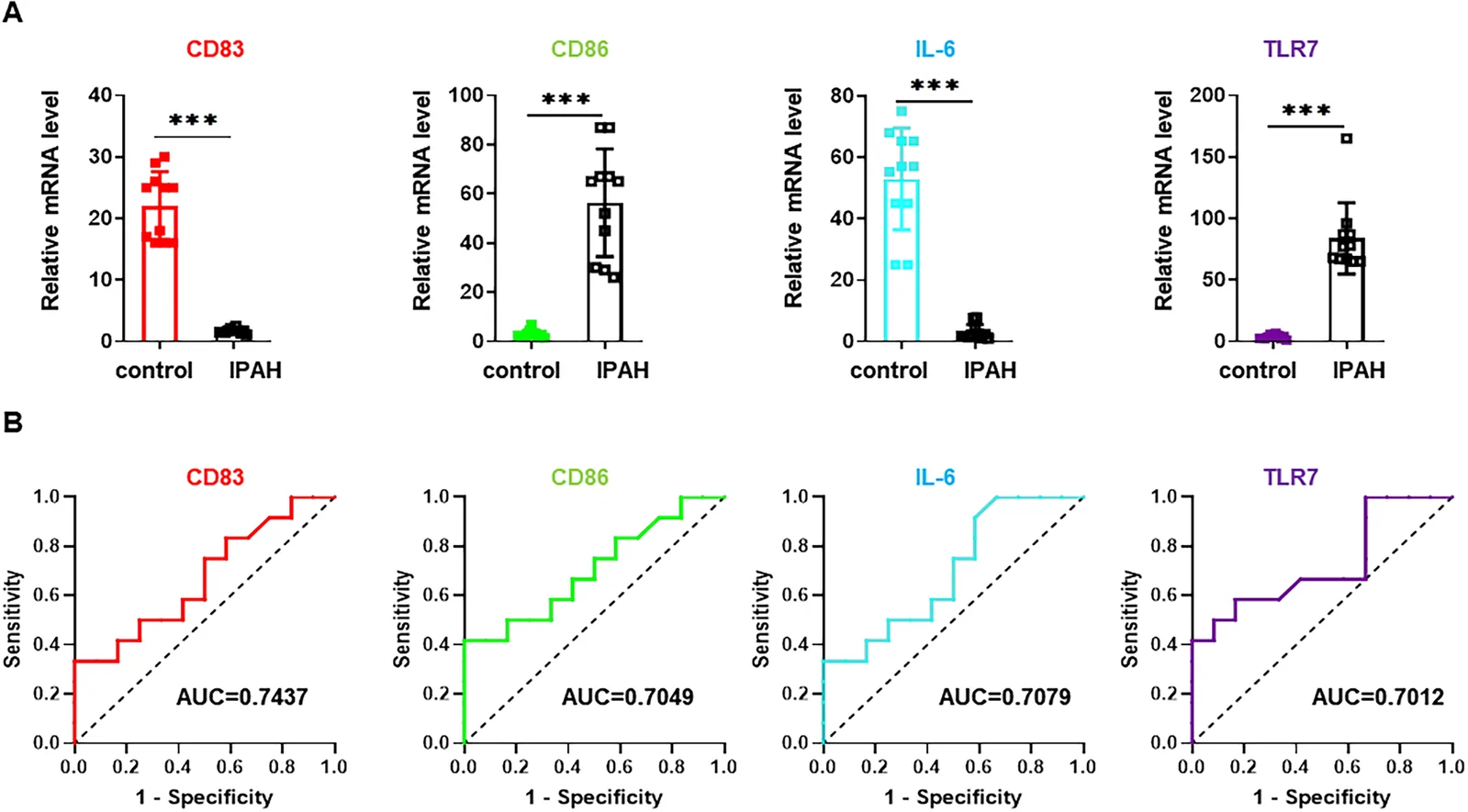
Biomarker validation in clinical IPAH peripheral blood mononuclear cell specimens. **(A)** Expression levels of CD86, TLR7, CD83 and IL-6 in clinical peripheral blood mononuclear cell (PBMC) samples. **(B)** ROC curve analysis of the diagnostic efficacy of CD86, TLR7, CD83 and IL-6 for IPAH. Data represent mean ± SEM. ***P < 0.001 compared to control, as analyzed by unpaired *t* test.

## Discussion

In this study, by integrating bulk lung transcriptomics, peripheral blood mononuclear cell transcriptomics, and single-cell RNA sequencing data, we systematically characterized the mechanobiology-associated transcriptomic remodeling landscape in pulmonary arterial hypertension. We constructed a mechanical-related gene set encompassing three dimensions—mechanical microenvironment, cytoskeleton and force generation, and mechanosensing signaling—and through weighted gene co-expression network analysis, machine learning-based prioritization, and single-cell localization analysis, ultimately identified an experimentally anchored four-gene panel (CD83, CD86, IL6, TLR7). The expression characteristics and diagnostic performance of these four genes were further validated in an MCT-induced pulmonary hypertension (PH) rat model with PAH-like lesions and PBMC samples isolated from IPAH patients.

The enrichment of stress-fiber, actomyosin and extracellular-matrix programs in our transcriptomic analyses places the candidate panel within a mechanically altered pulmonary vascular environment. Experimental work has shown that ECM stiffening can activate a YAP/TAZ-miR-130/301 feedback circuit that promotes further matrix remodeling in PH (10). This provides a rationale for examining immune-associated genes in a mechanobiology-informed screen. However, enrichment and co-expression do not establish the temporal order of mechanical and inflammatory events, and our study did not measure YAP/TAZ or RhoA/ROCK activity or perturb these pathways. The panel should therefore be interpreted as associated with this disease context, rather than as a demonstrated set of downstream targets of mechanical loading.

Immune activation in PAH does not require an ongoing infection. HIV-associated and schistosomiasis-associated PAH are recognized clinical entities, whereas idiopathic PAH is classified separately (1). Noninfectious vascular injury can also engage innate immunity through endogenous damage-associated molecular patterns (DAMPs). For example, Bauer et al. detected extracellular redistribution of HMGB1 in IPAH vascular lesions and showed that HMGB1 neutralization or TLR4 deletion attenuated hypoxia-induced PH in mice (5). This supports a role for injury-associated danger sensing in pulmonary vascular inflammation. Together with evidence for matrix-dependent vascular remodeling (10), it provides a plausible setting in which tissue injury, inflammatory signaling and altered mechanics may reinforce one another. The HMGB1 evidence concerns TLR4, however, and must not be treated as proof of TLR7 activation or of a direct mechanical pathway regulating our four-gene panel.

CD83 and CD86 provide a connection between antigen-presenting cells (APCs) and downstream immune responses, rather than evidence of infection. CD86 binds the stimulatory receptor CD28 and the inhibitory receptor CTLA-4 on T cells (14, 15). Its functional effect therefore depends on receptor engagement and the accompanying antigen-presentation context. CD83 has a distinct role: experimental studies show that membrane CD83 can oppose MARCH1-mediated ubiquitination and help maintain surface MHC class II and CD86 on dendritic cells (16). These mechanisms explain how APC-state changes could influence local immune responses following tissue injury. In our data, CD83/CD86 localized to myeloid/APC compartments, while CD83 decreased and CD86 showed compartment-dependent changes. Their divergent expression does not establish activation of the CD83-MARCH1 pathway, a uniform APC phenotype, or immune exhaustion. Cell composition, activation state and post-transcriptional regulation remain possible explanations. We did not measure surface costimulatory activity, antigen specificity or T-cell responses, so the contribution of these molecules to vascular inflammation remains a functional hypothesis.

IL-6 offers a more directly studied link between inflammatory signaling and pulmonary vascular remodeling. IL-6 binds membrane-bound or soluble IL-6 receptor (IL6R), engages gp130 and activates JAK/STAT3 signaling. Tamura et al. demonstrated increased membrane IL6R in PAH pulmonary artery smooth muscle cells and showed that IL6R signaling promoted their survival, whereas IL6R targeting reduced experimental PH (17). This mechanism provides a route by which inflammatory signals can support vascular-cell accumulation without requiring an infectious stimulus. Our lower IL6 expression in bulk lung and PBMC data, together with lower IL-6 protein in MCT-treated rat lungs, differs from reports of elevated circulating IL-6 in PAH (18, 19). These measurements address different biological compartments and do not quantify the same signaling process. Differences in cell composition, disease context, secretion and receptor availability could contribute, but were not resolved here. In particular, structural-cell enrichment of IL6 in the single-cell data does not demonstrate increased IL6 expression in those cells during disease. Because we did not measure IL6R, soluble IL6R or phosphorylated STAT3, our results cannot establish reduced IL-6 pathway activity, suppression of a protective IL-6 mechanism, or a general protective role for IL-6 in PAH.

TLR7 requires separate consideration because its expression does not establish either viral infection or receptor activation. TLR7 is an endosomal RNA sensor. In experimental systems, RNA-containing autoantigen immune complexes can deliver endogenous RNA to this compartment and induce TLR7-dependent interferon-alpha and IL-6 production (20). Downstream signaling involves MyD88-associated signaling proteins and IRF7-dependent type I interferon induction, alongside inflammatory signaling through NF-kappaB (21). Thus, recognition of appropriately delivered self-RNA provides a possible noninfectious route to TLR7 activation. The supporting evidence comes from immune-cell and autoimmunity studies; it does not demonstrate that this route operates in our PAH samples. We measured neither endogenous RNA ligands nor TLR7 pathway activity. In our data, TLR7 was down/non-significant in human bulk lung (logFC -0.448, P = 0.18), upregulated in public PBMCs (logFC +1.099, P = 8.1e-08), and sparsely detected by lung scRNA-seq. Removing TLR7 increased the independently standardized PBMC transfer AUC from 0.590 to 0.780, and its Shapley contribution was 0.073. Rat lung protein expression increased, further illustrating model- and compartment-dependent behavior. The TLR7 AUC of 0.850 pertains to the public PBMC analysis (**Figure 4G**), while the separate clinical PBMC analysis reported an AUC >0.7 (**Figure 8B**). These exploratory discrimination findings do not establish a pathogenic TLR7 mechanism in PAH.

The four-gene disease-state score achieved a leave-one-out cross-validation AUC of 0.765, and support vector machine sensitivity analysis yielded a concordant AUC of 0.722, indicating moderate diagnostic performance of this panel. Published PAH transcriptomic signatures have reported higher AUCs, but their validation designs vary. Duo et al. (22) reported AUCs of 0.948 and 0.945 in two cohorts; Shang et al. (23) reported approximately 0.95; Lian et al. (24) reported internal AUCs of 0.965 and 0.944 and external AUCs of 0.988 and 0.924; and Xiong et al. (25) reported AUCs of at least 0.969 in lung and 0.900 in PBMC data. Liu et al. (26) also performed external validation across blood and lung datasets with tissue-dependent performance. Direct comparison is limited by differences in tissue source, cohort composition, feature selection and validation strategy. We therefore interpret our leave-one-out cross-validated AUC of 0.765 as an internally cross-validated exploratory estimate from a small discovery cohort, not as evidence of generalizable clinical performance. Recent oncology gene-signature studies illustrate more clinically oriented prediction workflows: Saddozai et al. (27) combined LASSO selection with external validation and a lower-grade glioma prognostic nomogram, whereas their Ewing sarcoma study (28) used a Cox signature, external cohorts, nomogram construction and calibration. These studies predict cancer survival, so their metrics are not directly comparable with our PAH diagnostic AUC. Our mechanobiology-informed workflow instead prioritizes a cross-compartment PAH disease-state panel and reports internal discrimination only, without an individualized nomogram or prospective calibration. Its novelty lies in biological candidate prioritization rather than in a validated individual-level risk tool. In clinical PBMC samples, all four genes showed significant differences between IPAH patients and healthy controls, and the ROC AUC for each gene exceeded 0.7, supporting further assessment of their diagnostic potential. The cellular localization and known functions of these genes provide a rationale for investigating the immune-stromal environment in PAH (29). Their expression patterns alone cannot establish loss of immune tolerance, T-cell activation or a coordinated pathogenic pathway. Because no gene-knockdown, overexpression or mechanistic experiment was performed, therapeutic or precision-medicine implications remain hypothesis-generating. This score is not a clinically deployable nomogram or treatment-selection tool and requires independent prospective validation.

To further validate the expression characteristics and diagnostic value of the core genes (CD83, CD86, IL-6, and TLR7) identified by bioinformatics analysis and machine learning in pulmonary arterial hypertension, we established an MCT-induced pulmonary hypertension rat model and collected peripheral blood mononuclear cell samples from patients with idiopathic PAH and healthy controls for experimental validation. The results showed that compared with the control group, MCT-treated rats exhibited significantly elevated right ventricular systolic pressure, increased right ventricular hypertrophy, and markedly greater pulmonary arterial medial thickness, indicating successful establishment of the PAH animal model with typical pulmonary vascular remodeling and right ventricular dysfunction. At the protein level, CD86 and TLR7 expression was significantly upregulated in the lung tissues of model rats, while CD83 and IL-6 expression was significantly downregulated, although the TLR7 increase differed from its non-significant downward trend in human bulk lung transcriptomes. Further clinical validation in PBMC samples revealed that CD86 and TLR7 expression levels were significantly higher in IPAH patients than in healthy controls, whereas CD83 and IL-6 levels were significantly lower. The area under the ROC curve for all four genes exceeded 0.7, supporting their candidate diagnostic potential in this small validation cohort. Collectively, these experimental findings not only confirm the aberrant expression patterns of the core genes in PAH but also provide expression-level support for their potential as candidate diagnostic biomarkers, with any regulatory or therapeutic role remaining to be established in dedicated functional studies. These findings provide expression-level validation, with the cross-compartment inconsistencies described above.

This study has several limitations. First, the discovery cohort is small (31 lung samples, 18 PAH events; event-per-variable approximately 0.16 for the 111-feature screen). The machine-learning analysis is interpreted as candidate prioritization, and its internally cross-validated discrimination requires independent confirmation in larger cohorts. Second, the cross-sectional datasets provide disease-state contrasts and cannot establish temporal relationships or disease progression. Third, membership in the mechanobiology-informed gene set and expression associations do not demonstrate that the four genes are direct mechanosensors or downstream effectors of YAP/TAZ or RhoA/ROCK. Fourth, the analysis is limited to lung tissue, PBMCs and single-cell lung data; additional compartment-specific measurements could help resolve the observed heterogeneity.

The present study did not investigate microbial triggers, quantify endogenous danger ligands, or test the proposed signaling connections by pathway inhibition, gene perturbation or controlled mechanical stimulation. Consequently, immune-gene expression cannot establish infection, sterile ligand-driven activation, or causality. The DAMP, APC and IL-6 pathways discussed above represent literature-supported biological context, while a mechanical injury-self-RNA-TLR7 connection in PAH remains hypothetical. Studies combining defined stiffness or stretch conditions with ligand measurements, cell-specific perturbations and downstream signaling readouts would be needed to distinguish these possibilities.

## Conclusions

This study identified CD83, CD86, IL6, and TLR7 as immune-associated candidates through a mechanobiology-informed transcriptomic screen and expression-level validation. Their compartment-dependent behavior is compatible with altered immune-stromal interactions in PAH. Published evidence on endogenous danger sensing, antigen presentation and IL-6 signaling provides a biological rationale for immune involvement in noninfectious vascular injury. Our results do not establish that the four genes form a causal pathway or mediate the effects of mechanical stress. The panel remains an exploratory disease-state signature requiring independent clinical validation and targeted functional investigation.

## Data Availability

The original contributions presented in the study are included in the article/**Supplementary Material**. Further inquiries can be directed to the corresponding authors.

## References

[1] Humbert M Kovacs G Hoeper MM Badagliacca R Berger RMF Brida M . 2022 ESC/ERS guidelines for the diagnosis and treatment of pulmonary hypertension. Eur Respir J. (2023) 61:2200879. doi: 10.1183/13993003.00879-2022 36028254

[2] Rabinovitch M . Molecular pathogenesis of pulmonary arterial hypertension. J Clin Invest. (2012) 122:4306–13. doi: 10.1172/JCI60658 23202738 PMC3533531

[3] Thenappan T Ormiston ML Ryan JJ Archer SL . Pulmonary arterial hypertension: pathogenesis and clinical management. BMJ. (2018) 360:j5492. doi: 10.1136/bmj.j5492 29540357 PMC6889979

[4] Benza RL Gomberg-Maitland M Elliott CG Farber HW Foreman AJ Frost AE . Predicting survival in patients with pulmonary arterial hypertension: the REVEAL risk score calculator 2.0. Chest. (2019) 156:323–37. doi: 10.1016/j.chest.2019.02.004 30772387

[5] Bauer EM Shapiro R Zheng H Ahmad F Ishizawar D Comhair SA . High mobility group box 1 contributes to the pathogenesis of experimental pulmonary hypertension via activation of Toll-like receptor 4. Mol Med. (2012) 18:1509–18. doi: 10.2119/molmed.2012.00283 23269975 PMC3576475

[6] Dupont S Morsut L Aragona M Enzo E Giulitti S Cordenonsi M . Role of YAP/TAZ in mechanotransduction. Nature. (2011) 474(7350):179–83. doi: 10.1038/nature10137 21654799

[7] Humphrey JD Dufresne ER Schwartz MA . Mechanotransduction and extracellular matrix homeostasis. Nat Rev Mol Cell Biol. (2014) 15:802–12. doi: 10.1038/nrm3896 25355505 PMC4513363

[8] Waters CM Sporn PH Liu M Fredberg JJ . Cellular biomechanics in the lung. Am J Physiol Lung Cell Mol Physiol. (2002) 283:L503–9. doi: 10.1152/ajplung.00141.2002 12169567

[9] Tan W Madhavan K Hunter KS Park D Stenmark KR . Vascular stiffening in pulmonary hypertension: cause or consequence? (2013 Grover Conference series). Pulm Circ. (2014) 4:560–80. doi: 10.1086/677370 25610594 PMC4278618

[10] Bertero T Cottrill KA Lu Y Haeger CM Dieffenbach P Annis S . Matrix remodeling promotes pulmonary hypertension through feedback mechanoactivation of the YAP/TAZ-miR-130/301 circuit. Cell Rep. (2015) 13(5):1016–32. doi: 10.1016/j.celrep.2015.09.049 26565914 PMC4644508

[11] Langfelder P Horvath S . WGCNA: an R package for weighted correlation network analysis. BMC Bioinf. (2008) 9:559. doi: 10.1186/1471-2105-9-559 19114008 PMC2631488

[12] Friedman J Hastie T Tibshirani R . Regularization paths for generalized linear models via coordinate descent. J Stat Softw. (2010) 33:1–22. doi: 10.18637/jss.v033.i01 PMC2929880 20808728

[13] Stuart T Butler A Hoffman P Hafemeister C Papalexi E Mauck WM 3rd . Comprehensive integration of single-cell data. Cell. (2019) 177:1888–1902.e21. doi: 10.1016/j.cell.2019.05.031 31178118 PMC6687398

[14] Collins AV Brodie DW Gilbert RJ Iaboni A Manso-Sancho R Walse B . The interaction properties of costimulatory molecules revisited. Immunity. (2002) 17:201–10. doi: 10.1016/S1074-7613(02)00362-X 12196291

[15] Chen L Flies DB . Molecular mechanisms of T cell co-stimulation and co-inhibition. Nat Rev Immunol. (2013) 13:227–42. doi: 10.1038/nri3405 23470321 PMC3786574

[16] Tze LE Horikawa K Domaschenz H Howard DR Roots CM Rigby RJ . CD83 increases MHC II and CD86 on dendritic cells by opposing IL-10-driven MARCH1-mediated ubiquitination and degradation. J Exp Med. (2011) 208:149–65. doi: 10.1084/jem.20092203 21220452 PMC3023131

[17] Tamura Y Phan C Tu L Le Hiress M Thuillet R Jutant EM . Ectopic upregulation of membrane-bound IL6R drives vascular remodeling in pulmonary arterial hypertension. J Clin Invest. (2018) 128:1956–70. doi: 10.1172/JCI96462 29629897 PMC5919830

[18] Humbert M Monti G Brenot F Sitbon O Portier A Grangeot-Keros L . Increased interleukin-1 and interleukin-6 serum concentrations in severe primary pulmonary hypertension. Am J Respir Crit Care Med. (1995) 151:1628–31. doi: 10.1164/ajrccm.151.5.7735624 7735624

[19] Soon E Holmes AM Treacy CM Doughty NJ Southgate L Machado RD . Elevated levels of inflammatory cytokines predict survival in idiopathic and familial pulmonary arterial hypertension. Circulation. (2010) 122:920–7. doi: 10.1161/circulationaha.109.933762 20713898

[20] Savarese E Chae OW Trowitzsch S Weber G Kastner B Akira S . U1 small nuclear ribonucleoprotein immune complexes induce type I interferon in plasmacytoid dendritic cells through TLR7. Blood. (2006) 107:3229–34. doi: 10.1182/blood-2005-07-2650 16368889

[21] Uematsu S Sato S Yamamoto M Hirotani T Kato H Takeshita F . Interleukin-1 receptor-associated kinase-1 plays an essential role for Toll-like receptor (TLR)7- and TLR9-mediated interferon-alpha induction. J Exp Med. (2005) 201:915–23. doi: 10.1084/jem.20042372 15767370 PMC2213113

[22] Duo M Liu Z Zhang Y Li P Weng S Xu H . Construction of a diagnostic signature and immune landscape of pulmonary arterial hypertension. Front Cardiovasc Med. (2022) 9:940894. doi: 10.3389/fcvm.2022.940894 36531729 PMC9751874

[23] Shang Z Sun J Hui J Yu Y Bian X Yang B . Construction of a support vector machine-based classifier for pulmonary arterial hypertension patients. Front Genet. (2021) 12:781011. doi: 10.3389/fgene.2021.781011 34880909 PMC8647811

[24] Lian G You J Lin W Gao G Xu C Wang H . Bioinformatics analysis of the immune cell infiltration characteristics and correlation with crucial diagnostic markers in pulmonary arterial hypertension. BMC Pulm Med. (2023) 23:300. doi: 10.1186/s12890-023-02584-4 37582718 PMC10428559

[25] Xiong P Huang Q Mao Y Qian H Yang Y Mou Z . Identification of an immune-related gene panel for the diagnosis of pulmonary arterial hypertension using bioinformatics and machine learning. Int Immunopharmacol. (2025) 144:113694. doi: 10.1016/j.intimp.2024.113694 39616855

[26] Liu H Wu M Qi G Ma F Cao Y . Identification and validation of potential diagnostic biomarkers for pulmonary arterial hypertension based on gene expression profiling. Pulm Circ. (2025) 15:e70207. doi: 10.1002/pul2.70207 41221472 PMC12598573

[27] Saddozai UAK Lu Z Dong S Khawar MB Fan Z Cai L . Prognostic value of metal-based ferroptosis and cuproptosis genes and score in lower grade gliomas. Front Immunol. (2025) 16:1608077. doi: 10.3389/fimmu.2025.1608077 40963614 PMC12436455

[28] Biele E Schober SJ Prexler C Thiede M Heyking KV Gassmann H . Integrative analysis of T cell-associated markers in Ewing sarcoma reveals prognostic signatures and immune dynamics. Front Immunol. (2025) 16:1586544. doi: 10.3389/fimmu.2025.1586544 40607387 PMC12213708

[29] Price LC Wort SJ Perros F Dorfmüller P Huertas A Montani D . Inflammation in pulmonary arterial hypertension. Chest. (2012) 141:210–21. doi: 10.1378/chest.11-0793 22215829

