# Supplementary material for: Integrated multi-omics and machine learning identify core mechanobiology-associated immune genes in pulmonary arterial hypertension
Source: Front Immunol. 2026 Oct 6;17:1867114. doi: 10.3389/fimmu.2026.1867114 (PMC13680207; doi:10.3389/fimmu.2026.1867114)

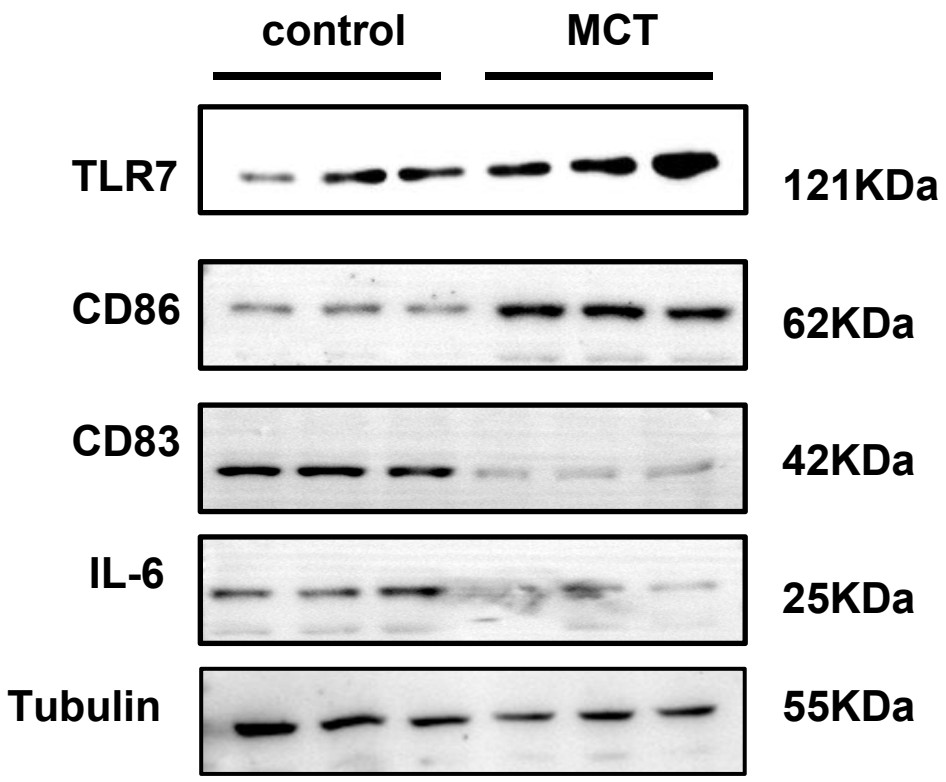

TLR7

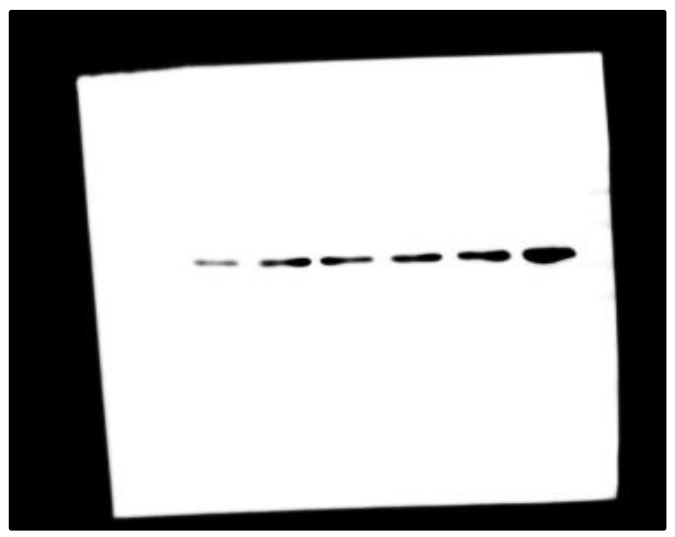

CD86

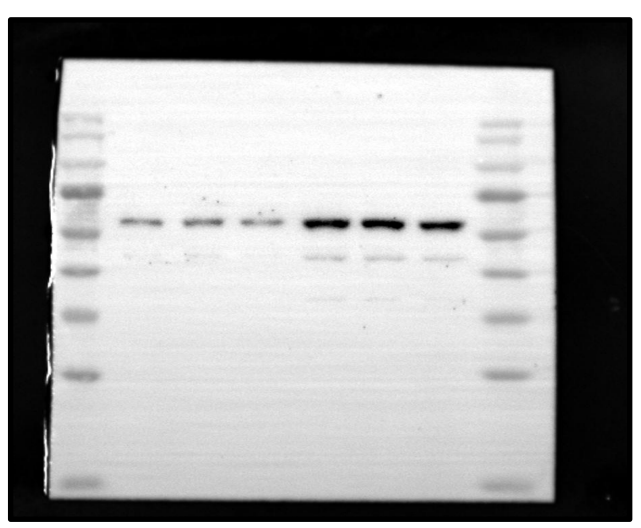

CD83

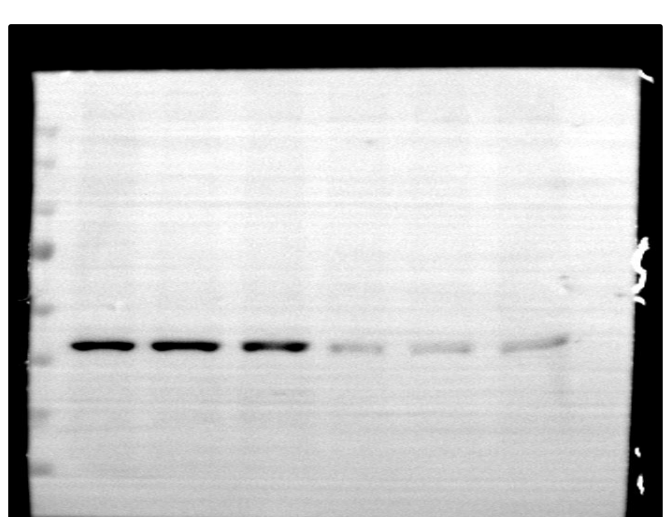

IL-6

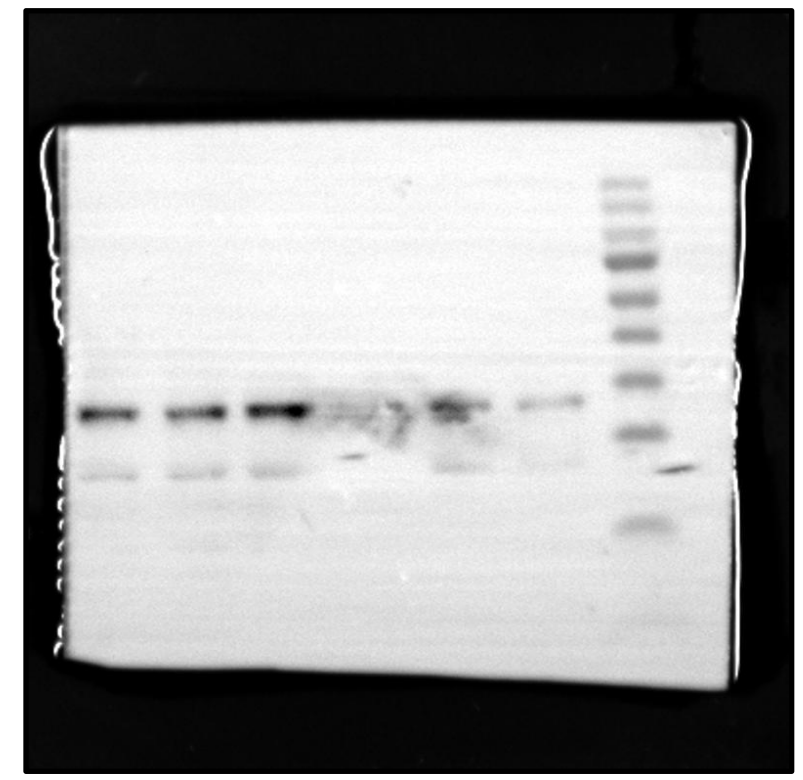

Tubulin

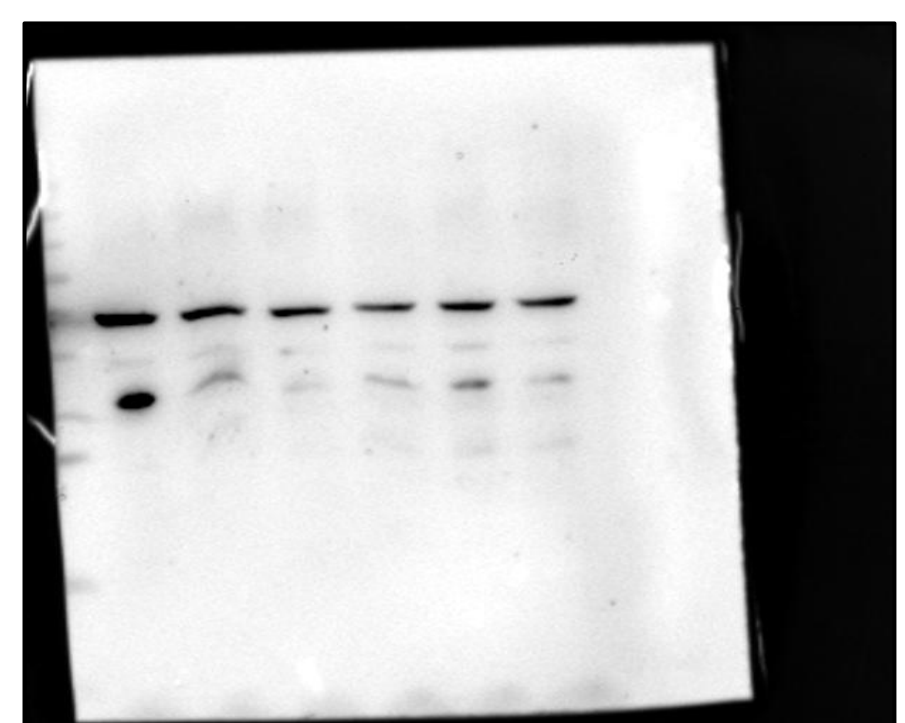

Supplement: Supplementary file 1 [file DataSheet1.pdf]
