# Supplementary material for: Integrated multi-omics and machine learning identify core mechanobiology-associated immune genes in pulmonary arterial hypertension
Source: Front Immunol. 2026 Oct 6;17:1867114. doi: 10.3389/fimmu.2026.1867114 (PMC13680207; doi:10.3389/fimmu.2026.1867114)
Supplement: Supplementary file 2 [file Table1.docx]

Figure S1. Supplementary candidate-prioritization and model-sensitivity analyses.

(A) Univariate diagnostic performance of candidate genes. (B) Candidate-gene expression heatmap. (C) Candidate-gene correlation structure. (D) Overlap between LASSO- and random-forest-selected stable genes. (E) LASSO cross-validation deviance. (F) Random-forest out-of-bag error. These panels provide model-transparency and sensitivity context for Fig. 4.

Figure S2. Exploratory myeloid/APC cell-state analysis.

(A) Myeloid/APC subcluster UMAP. (B) Cell-state coordinate inferred within the myeloid/APC compartment. (C) Subcluster composition by group. (D) Coordinate-density distribution. (E) Canonical marker dot plot. (F) Four-gene expression across myeloid/APC subclusters. (G) Expression of CD83, CD86, IL6 and TLR7 along the coordinate. The analysis is interpreted as a phenotypic gradient, not as a developmental trajectory.
